# Multilayer omics reveals the molecular mechanism of early infection of *Clonorchis sinensis* juvenile

**DOI:** 10.1186/s13071-023-05891-1

**Published:** 2023-08-16

**Authors:** Yuhong Wu, Xueling Deng, Zhanshuai Wu, Dengyu Liu, Xiaoyin Fu, Lili Tang, Shanshan He, Jiahui Lv, Jilong Wang, Qing Li, Tingzheng Zhan, Zeli Tang

**Affiliations:** 1 Department of Cell Biology and Genetics, School of Basic Medical Sciences, Guangxi Medical University, Nanning, 530021 China; 2 Department of Immunology, Guangxi University of Chinese Medicine, Nanning, 530021 China; 3 Guangxi Key Laboratory of Translational Medicine for Treating High-Incidence Infectious Diseases With Integrative Medicine, Nanning, 530021 China; 4 Department of Parasitology, School of Basic Medical Sciences, Guangxi Medical University, Nanning, 530021 China; 5 Key Laboratory of Longevity and Aging-Related Diseases of Chinese Ministry of Education, Guangxi Medical University, Nanning, 530021 China; 6 Key Laboratory of Basic Research on Regional Diseases (Guangxi Medical University), Education Department of Guangxi Zhuang Autonomous Region, Nanning, 530021 China

**Keywords:** *Clonorchis sinensis*, Early infection, Host, Interaction, Liver injury, Molecular mechanism, Multilayer omics

## Abstract

**Background:**

Clonorchiasis remains a non-negligible global zoonosis, causing serious socioeconomic burdens in endemic areas. *Clonorchis sinensis* infection typically elicits Th1/Th2 mixed immune responses during the course of biliary injury and periductal fibrosis. However, the molecular mechanism by which *C. sinensis* juvenile initially infects the host remains poorly understood.

**Methods:**

The BALB/c mouse model was established to study early infection (within 7 days) with *C. sinensis* juveniles. Liver pathology staining and observation as well as determination of biochemical enzymes, blood routine and cytokines in blood were conducted. Furthermore, analysis of liver transcriptome, proteome and metabolome changes was performed using multi-omics techniques. Statistical analyses were performed using Student's t-test.

**Results:**

Histopathological analysis revealed that liver injury, characterized by collagen deposition and inflammatory cell infiltration, occurred as early as 24 h of infection. Blood indicators including ALT, AST, WBC, CRP and IL-6 indicated that both liver injury and systemic inflammation worsened as the infection progressed. Proteomic data showed that apoptosis and junction-related pathways were enriched within 3 days of infection, indicating the occurrence of liver injury. Furthermore, proteomic and transcriptomic analysis jointly verified that the detoxification and antioxidant defense system was activated by enrichment of glutathione metabolism and cytochrome P450-related pathways in response to acute liver injury. Proteomic-based GO analysis demonstrated that biological processes such as cell deformation, proliferation, migration and wound healing occurred in the liver during the early infection. Correspondingly, transcriptomic results showed significant enrichment of cell cycle pathway on day 3 and 7. In addition, the KEGG analysis of multi-omics data demonstrated that numerous pathways related to immunity, inflammation, tumorigenesis and metabolism were enriched in the liver. Besides, metabolomic screening identified several metabolites that could promote inflammation and hepatobiliary periductal fibrosis, such as CA7S.

**Conclusions:**

This study revealed that acute inflammatory injury was rapidly triggered by initial infection by *C. sinensis* juveniles in the host, accompanied by the enrichment of detoxification, inflammation, fibrosis, tumor and metabolism-related pathways in the liver, which provides a new perspective for the early intervention and therapy of clonorchiasis.

**Graphical Abstract:**

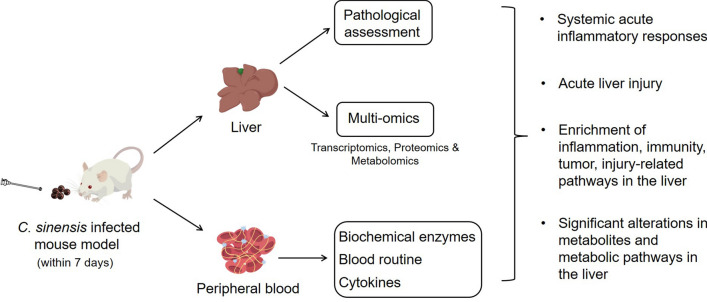

**Supplementary Information:**

The online version contains supplementary material available at 10.1186/s13071-023-05891-1.

## Background

Clonorchiasis, caused by *Clonorchis sinensis*, remains a global public health problem that cannot be neglected with a wide epidemic range and a huge number of infections [1, 2]. Currently, an estimated 15 million people worldwide are infected, mainly distributed in East and Southeast Asia, notably in China, South Korea and northern Vietnam [2]. Recent investigations demonstrated that the prevalence of *C. sinensis* infection remains high in endemic areas. The prevalence rate was reported to be as high as 60.3% and 28.9% in Hengxian and Binyang counties in southern China, respectively [3, 4]. It is documented that the positive rate of *C. sinensis* among residents along the five major rivers in South Korea was up to 8.4% [5]. One study shows a high epidemic rate of *C. sinensis* (40.4%) in rural communities in Yen Bai and Thanh Hoa provinces, northern Vietnam [6]. In addition, the reinfection rate of *C. sinensis* in endemic areas is verified to be alarmingly high [7].

*Clonorchis sinensis* adults mainly parasitize in human hepatobiliary ducts and induce different clinical symptoms. The mild ones show no obvious symptoms; the severe ones show fever, diarrhea, hepatomegaly, jaundice and other symptoms and can also cause a variety of complications such as cholecystitis, cholangitis, cholelithiasis, liver cirrhosis and even cholangiocarcinoma [1, 8]. In 2009, *C. sinensis* was clearly classified as a Group I biological carcinogen causing cholangiocarcinoma by the World Health Organization (WHO) [9]. The severe morbidity of hepatobiliary duct and recurrent susceptibility caused by *C. sinensis* have brought both serious disease and economic burden to endemic areas and countries. The WHO estimates that the global burden of clonorchiasis in 2010 was 522,863 disability-adjusted life years (DALYs) [10]. Zhao et al. evaluated that the DALYs of clonorchiasis in China in 2016 were up to 489,174.04 [11]. Therefore, the global harm caused by clonorchiasis remains severe, the pathogenesis of *C. sinensis* should be further clarified, and effective prevention and control strategies need to be formulated as soon as possible.

*Clonorchis sinensis* infection elicits immunoinflammatory changes, progressive peribiliary fibrosis and hyperplasia of biliary epithelial cells in the host, mainly due to mechanical damage and excretory-secretory products (ESPs) of worms [12, 13]. During *C. sinensis* parasitism, massive compounds are secreted to trigger complex immune responses in the host, which are dominated by type 1 responses in the juvenile stage and type 2 responses in the adult stage [13, 14]. However, little is known about the physiological responses and pathological manifestations caused by early infection by *C. sinensis* juveniles in the host. In this study, we employed a range of techniques including histopathological assessment, biochemical analysis, cytokine assays and multi-omics approaches to investigate the dynamic changes in blood indicators, liver pathology, gene transcriptional expression and metabolic state in a mouse model of *C. sinensis* infection. Our objective was to elucidate the pathogenic mechanisms by which *C. sinensis* infects the host during the early stages of infection.

## Methods

### Animals and parasites

SPF female BALB/c mice (6 weeks old, 17–21 g) were purchased from the Hunan SJA Laboratory Animal Co., Ltd. All the animals were raised well in a temperature-controlled room (23 °C ± 2 °C) with a 12 h dark/light cycle and fed on standard diet.

*Clonorchis sinensis* metacercariae were collected from naturally infected freshwater fish of *Pseudorasbora parva* in Hengxian county, Guangxi Zhuang Autonomous Region, China. Living metacercariae were obtained by digesting fish with optimized artificial gastric juice (pH 2.0, 0.9% NaCl, 0.8% pepsin) [15]. Briefly, the minced fish incubated in digestive juice was shaken overnight at 37 °C and 150 rpm. Then, the suspension was filtered through a 60–80 mesh sieve to remove indigestible residues and rinsed with distilled water three to four times until the supernatant in the triangular beaker was clear. Finally, the living metacercariae were picked up under an optical microscope and stored in PBS at 4 °C.

### Animal infection and sample collection

A total of 29 BALB/c mice were first acclimatized for 1 week before starting the experiment. The mice were randomly divided into the following six groups: 0 h (0 h, control) group, 6 h (6 h) group, 18 h (18 h) group, 24 h (24 h) group, 3 days (3 d) group and 7 days (7 d) group. Except for four mice in the 18 h group (*n* = 4), each of the other groups consisted of five mice (*n* = 5). All mice, except for control mice, were infected with living metacercariae (60 metacercariae/200 μl PBS per mouse) by intragastric administration. The control mice received an equivalent volume of PBS (200 μl) by gavage.

**Fig. 1:**
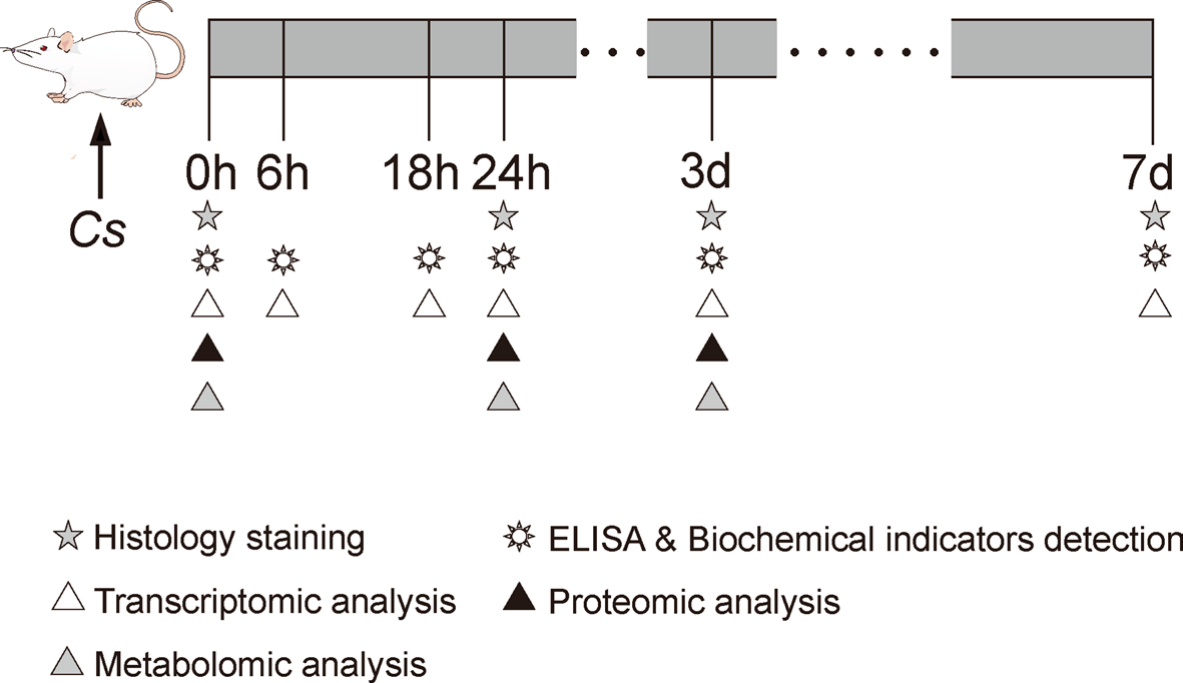
Schematic diagram of sample collection and detection of *C. sinensis*-infected mice

The mice were killed at 0 h, 6 h, 18 h, 24 h, 3 d and 7 d post intragastric administration to collect corresponding samples. First, peripheral blood samples were collected to preparate serum and anticoagulant whole blood, respectively. Serum samples were used for cytokine assay by ELISA. The anticoagulated blood was used to measure blood routine indexes and the proportion of CD4^+^ T and CD8^+^ T lymphocytes, respectively. Macroscopic photographs were taken for the livers from the mice in each group. The left lobe of each liver was then divided into quadruplicates, with one piece fixed in 4% paraformaldehyde for histopathological staining and the other three snap-frozen in liquid nitrogen for RNA sequencing, proteomics analysis and metabolomics analysis, respectively. Strategy chart of sample collection and index detection is shown in Fig. 1.

### Histology staining

Following death, liver tissues of mice were collected from the middle of the hepatic left lobe, fixed in 4% paraformaldehyde and embedded in paraffin. The tissues were then cut into 5-μm sections, and we conducted hematoxylin and eosin (H&E) staining and Masson’s trichrome staining, respectively. The stained sections were observed and evaluated under the optical microscope. Liver fibrosis in each group was evaluated morphologically using the Ishak fibrosis score [16], and the area of collagen staining was quantified with Image J software.

### Biochemical indicator detection

The blood samples of mice from each time point were collected and prepared for serum and anticoagulant blood. Serum hepatic enzyme activities were determined using alanine aminotransferase (ALT) assay kit and aspartate aminotransferase (AST) assay kit (Jiancheng, Nanjing, China), respectively. The levels of white blood cells (WBC), lymphocytes (LYM), granulocytes (GRAN), monocytes (MONO), red blood cells (RBC), hematocrit (HCT), hemoglobin (HGB) and platelet (PLT) were determined using an automatic blood analyzer (Servicebio, Wuhan, China).

### Enzyme-linked immunosorbent assay (ELISA)

To examine the immune responses, the levels of cytokines in serum, including IL-6, IL-1β, TNF-ɑ, IL-4 and IL-10, were quantified by corresponding ELISA kits according to the manufacturer's instructions (Thermo Fisher Scientific, Waltham, MA, USA). In addition, the levels of C-reactive protein (CRP) in serum samples were determined using mouse CRP ELISA kit (MultiSciences, Hangzhou, China).

### Flow cytometry

To evaluate the proportion of CD4^+^ and CD8^+^ T cells, the anticoagulated whole blood samples of group 0 h, 24 h, 3 d and 7 d were collected to prepare single-cell suspension. Afterwards, cells were phenotypically analyzed by flow cytometry (BD, Franklin Lakes, NJ, USA) after using the anti-mouse monoclonal antibodies of CD4-Percp, CD8-APC and CD3-PE (BD, Franklin Lakes, USA) for surface staining.

### RNA sequencing

To evaluate gene expression levels, total RNA was extracted from liver tissue of three mice from each group by RNA extraction kit (DP761, Tiangen, Beijing, China) for quantitative and qualitative analysis [17]. Transcriptome sequencing was performed on the BGISEQ platform by MONITOR HELIX Biotechnology (Shanghai, China). Quality control was performed on the raw data with fastp software v0.23.0. The clean data were subsequently aligned to the mouse genome reference sequence. The RPKM (reads per kilobase per million reads) was used as a measure of gene expression [18]. Finally, transcript information was analyzed using public data base gene ontology (GO) and Kyoto Encyclopedia of Genes and Genomes (KEGG).

### Quantitative real-time PCR (qPCR)

According to the instructions, the total RNA of mouse liver tissue was extracted using the Animal Total RNA Isolation Kit (Foregene, Sichuan, China). The cDNA was generated using *TransScript*^®^ Uni All-in-One First-Strand cDNA Synthesis SuperMix for qPCR (One-Step gDNA Removal) (TransGen, Beijing, China). QPCR was performed using the StepOnePlus^™^ Real-Time fluorescent quantitative PCR system (Thermo Fisher Scientific, Waltham, MA, USA). Gene expression levels were normalized to the housekeeping gene β-actin. The primer sequences of Col1a1, Col1a2, Spp1, Hmmr, Cdc20 and Ccnb2 used in the qPCR analysis were given in Additional file 1: Table S1.

### Proteomic analyses

To explore the protein expression levels, mouse liver tissues from four groups of 0 h, 6 h, 24 h and 3 d were extracted. For each time point, three mice were used from each group. Tissue proteins were extracted with RIPA lysis buffer (Beyotime, Shanghai, China) [19]. The samples were run on Q Exactive HF mass spectrometer (ThermoFisher Scientific, Waltha, MA, USA), and the raw data were searched using MaxQuant platform (https://maxquant.org/). The search results were then imported into Skyline software to generate the spectral library used for data independent acquisition (DIA) analysis. For DIA, the method consisted of a full MS1 scan (400–1200 m/z, resolution 45,000, maximum injection time 35 ms, AGC target 1E6) followed by 31 DIA windows (resolution 15,000, AGC target 1E5, maximum injection time of auto). NCE (normalized collision energy) was set to 28. The data were acquired by MONITOR HELIX Biotechnology (Shanghai, China).

The identified proteins were classified by GO annotation, including biological process (BP), cellular component (CC) and molecular function (MF). The KEGG database was used to identify the enriched functional pathways.

### LC-MS/MS-based untargeted metabolomics analyses

Liver tissues were prepared from three groups of 0 h, 24 h and 3 d for metabolite detection using untargeted liquid chromatography tandem mass spectrometry (LC-MS/MS). Sample preparation was performed as previously reported with minor modifications [20, 21]. Briefly, samples were injected into Dionex Ultimate 3000 UHPLC (Dionex, Sunnyvale, CA, USA) and run on a poroshell 120 EC-C18 reversed phase column. Detection was carried out on a Q Exactive HF Hybrid Quadrupole-Orbitrap mass spectrometer (Thermo Fisher Scientific, Waltham, MA, USA) with data acquisition executed using Xcalibur 3.1 software. Samples were analyzed by positive and negative electrospray ionization (ESI +/−) Full-MS scan mode (resolution 60,000, maximum injection time 200 ms, spray voltages 2.7 and 3.6 kV for negative and positive modes, respectively). ESI +/−data-dependent MS2 spectra were generated for samples at resolution, 15,000 FWHM; maximum injection time, 50 ms; isolation window, 2.0 m/z. The metabolomics data were acquired and analyzed by MONITOR HELIX Biotechnology (Shanghai, China), including differential metabolite analysis, significant difference metabolite screening and KEGG pathway enrichment analysis of differential metabolites.

### Statistical analyses

All results were represented as mean ± standard deviation (SD). SPSS 23.0 software was used for data analysis. Student’s t-test was employed to analyze histological, biochemical and qPCR data. For transcriptomic data, significantly enriched items in differentially expressed genes (DEGs) were tested with hypergeometric distribution. For proteomic data, the number of differentially expressed protein (DEP) (a), number of all quantitative protein (b) in each GO/KEGG term, total number of DEP (c) annotated by GO/KEGG terms and total number of all quantitative protein (d) annotated by GO/KEGG terms were calculated, and then the four numbers (a, b, c, d) were used to calculate Fisher' exact test *P* values and enrichment folds. Fold enrichment = (a/c)/(b/d). For metabolomic data, FC analysis and t-test/nonparametric test were employed to analyze the difference between two groups of samples. VIP values of metabolites were obtained using orthogonal partial least squares discrimination analysis (OPLS-DA). Metabolic pathway analysis was carried out using the well-established mummichog algorithm. *P* < 0.05 was considered statistically significant.

## Results

### Gross and histopathological changes of mouse liver caused by *C. sinensis* infection

**Fig. 2:**
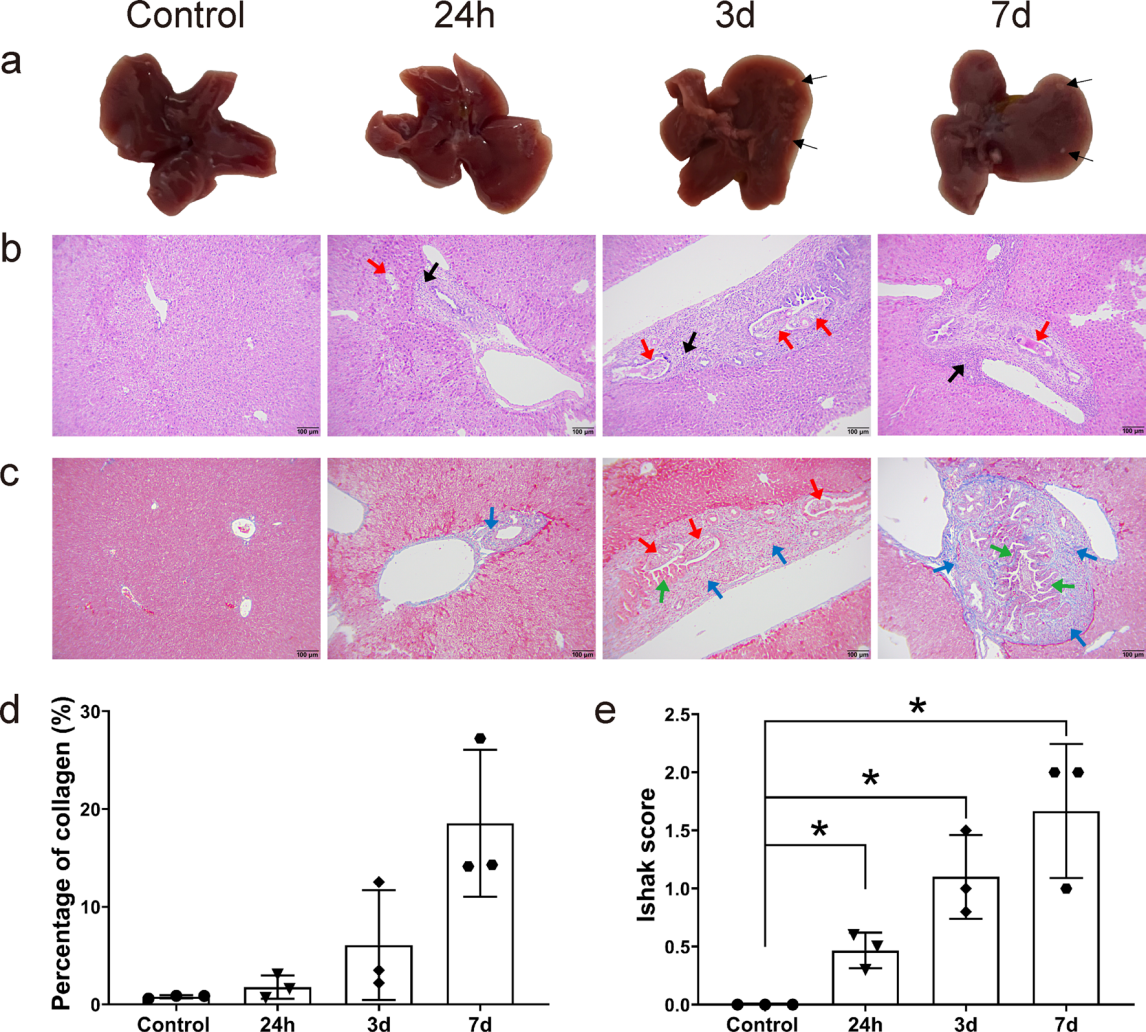
Gross observation and histopathological changes of liver in mice infected with *Clonorchis sinensis* at different time points. **a** Gross morphology of livers. Inflammatory nodules are marked by thin black arrows. **b** Liver sections of *C. sinensis*-infected mice were stained with H&E staining (× 100). Red arrows and heavy black arrows represent adult worms and inflammatory cell infiltration, respectively. **c** Liver sections were prepared for Masson trichrome staining (× 100). Red, green and blue arrows indicate *C. sinensis* juvenile, biliary epithelial hyperplasia and collagen deposition, respectively. **d** Percentage of collagen fiber area in liver tissue. **e** Ishak score of Masson staining. Data are shown as mean ± SD (*n* = 3). *P < 0.05

On day 3 post *C. sinensis* infection, 1–2 white nodules (thin black arrows) were observed on the liver surface of mice, and similar white foci (thin black arrows) were also found in the 7 d group (Fig. 2a).

Histologically, juvenile worms (red arrows) were found in liver tissue staining sections of 24 h, 3 d and 7 d groups (Fig. 2b and c). The phenomena of inflammatory cell infiltration (thick black arrows), biliary epithelium hyperplasia (green arrows) and collagen deposition around the bile duct (blue arrows) were first observed in the 24 h group and more pronounced in the 3 d and 7 d groups (Fig. 2b and c). The area of collagen deposition increased gradually as the duration of infection progressed (Fig. 2d). In addition, compared with the control group, all the infection groups showed a significant increase in Ishak score (24 h vs. 0 h: *t*_(4)_ = -5.29, *P* = 0.034, 3d vs. 0 h: *t*_(4)_ = – 5.284, *P* = 0.034, 7d vs. 0 h: *t*_(4)_ = – 5, *P* = 0.038, Fig. 2e).

### Changes of biochemical enzymes, blood routines, cytokines and T lymphocyte proportions in blood after *C. sinensis* infection

**Fig. 3:**
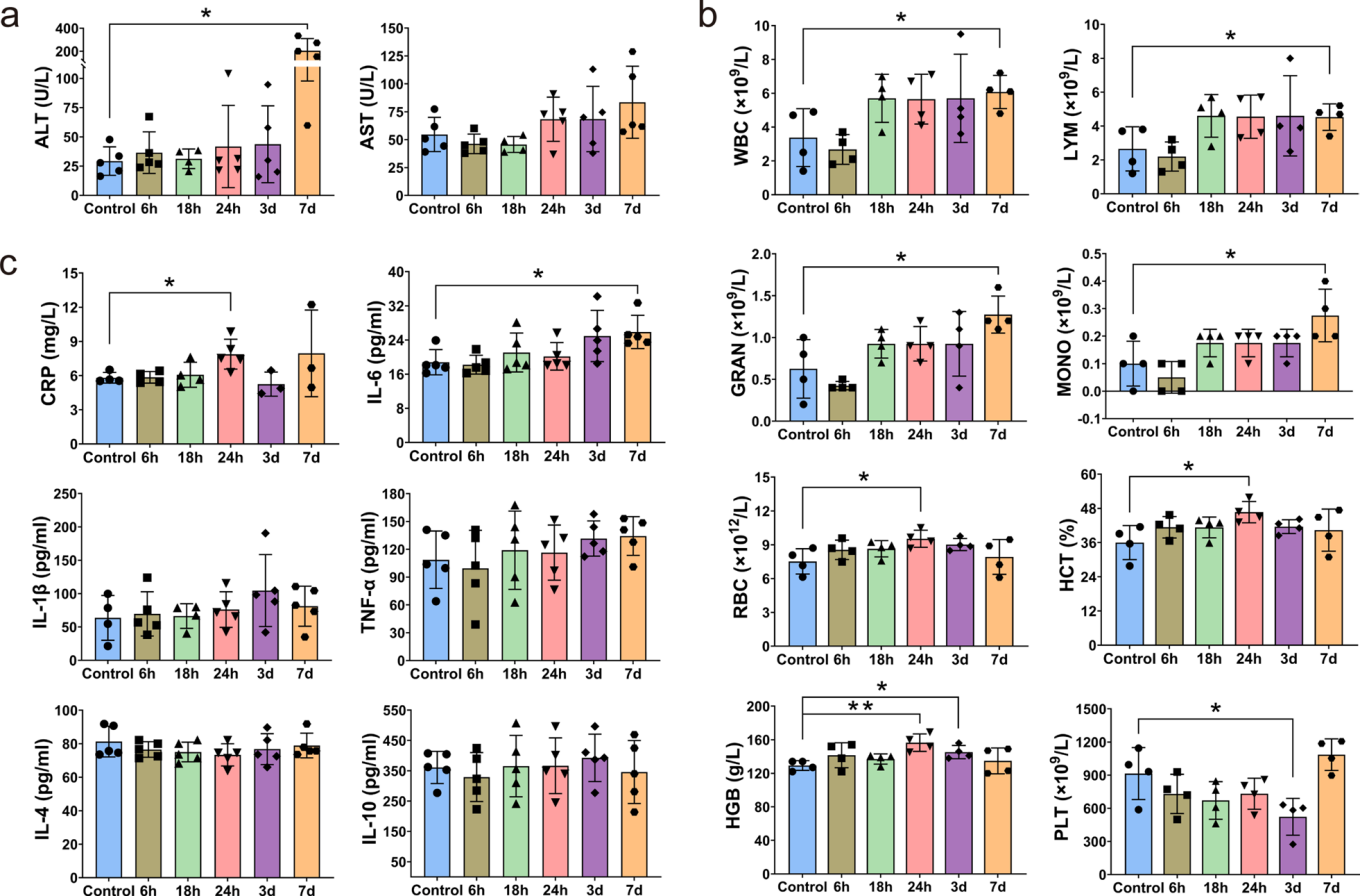
Levels of serum biochemical enzymes, blood routine indexes and serum cytokines. The mice were killed at 0 h, 6 h, 18 h, 24 h, 3 d and 7 d to prepare corresponding serum or anticoagulation samples for detection of the activities of ALT and AST (**a**), content of WBC, LYM, GRAN, MONO, RBC, HCT, HGB and PLT (**b**) and levels of CRP, IL-6, IL-1β, TNF-ɑ, IL-4 and IL-10 (**c**), respectively. Each symbol in each graph represents a sample. Data are presented as mean ± SD. *P < 0.05, **P < 0.01

The serological tests showed an increase in both ALT and AST levels from 24 h of infection, with ALT levels significantly elevated on day 7 (*t*_(8)_ = −3.656, *P* = 0.021, Fig. 3a). Blood routine data demonstrated that the number of WBC, LYM, GRAN and MONO increased at all infection time points except for 6 h and reached the highest point on the 7th day (WBC: *t*_(6)_ = −2.740, *P* = 0.034, LYM: *t*_(6)_ = −2.470, *P* = 0.048, MONO: *t*_(6)_ = −2.782, *P* = 0.032, GRAN: *t*_(6)_ = −3.138, *P* = 0.02, Fig. 3b). The blood indexes of RBC, HCT and HGB increased at all time points, among which RBC and HCT increased significantly at 24 h of infection (RBC: *t*_(6)_ = −2.967, *P* = 0.025, HCT: *t*_(6)_ = −3.028, *P* = 0.023), while HGB increased significantly at both 24 h and 3 d (HGB: 24 h vs. 0 h: *t*_(6)_ = −4.586, *P* = 0.004, 3d vs. 0 h:* t*_(6)_ = −3.258, *P* = 0.017, Fig. 3b). However, except for 7 d, the number of PLT decreased at all the other infection points, especially on 3 d (*t*_(6)_ = 2.717, *P* = 0.035, Fig. 3b).

The results of ELISA detection showed that, compared with the 0 h group, the levels of serum acute inflammatory reaction protein CRP significantly increased at 24 h post infection (*t*_(7)_ = −2.97, *P* = 0.021, Fig. 3c). The levels of pro-inflammatory cytokines (IL-6, IL-1β and TNF-α) in serum increased with the extension of infection time, and the level of IL-6 significantly increased on the 7th day of infection (*t*_(8)_ = −3.246, *P* = 0.012, Fig. 3c). However, no significant changes of anti-inflammatory cytokines (IL-4 and IL-10) were detected (Fig. 3c). Additionally, the results of flow cytometry verified that the proportions of CD4^+^ and CD8^+^ T cells in peripheral blood showed a fluctuating trend (Additional file 2: Fig. S1). The proportion of CD4^+^ T cells decreased at 24 h of infection, increased on 3 d and then decreased again on 7 d (Additional file 2: Fig. S1b), while the trend of CD8^+^ T cells was exactly the opposite (Additional file 2: Fig. S1c). Therefore, the ratio of CD4^+^/CD8^+^ T cells also presented a fluctuating trend (Additional file 2: Fig. S1d).

### DEGs in mouse liver induced by *C. sinensis* infection

**Fig. 4:**
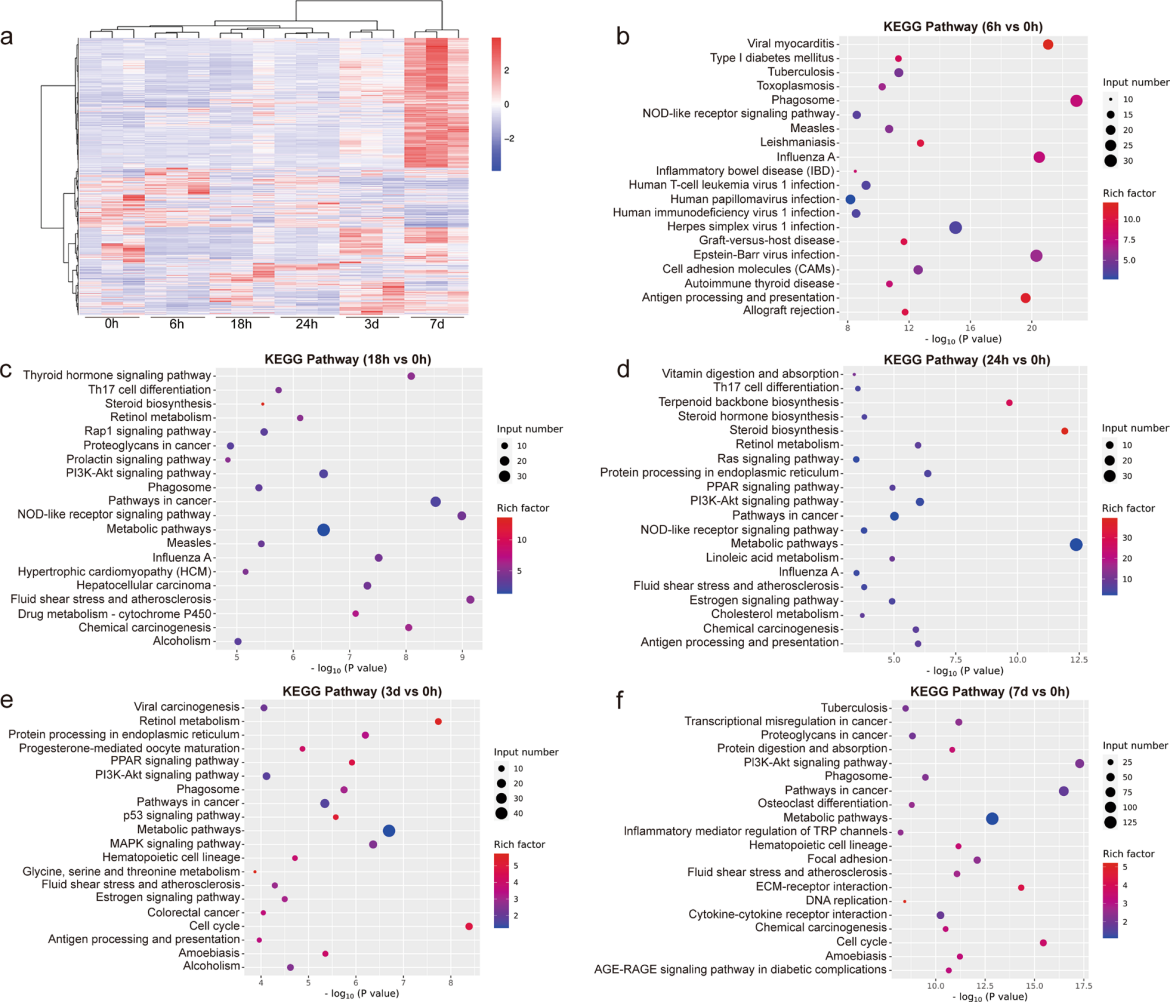
Comparative analysis of DEGs and changes of main signaling pathways in mouse liver induced by *Clonorchis sinensis* infection at different time points. **a** Heatmap and hierarchical clustering of DEGs. Red and blue represent genes up- and downregulated, respectively. **b**–**f** KEGG pathway enrichment analysis of the identified DEGs between 6 and 0 h groups, 18 h and 0 h groups, 24 h and 0 h groups, 3 d and 0 h groups and 7 d and 0 h groups, respectively. The top 20 most-enriched pathways are shown

To elucidate the effects of early *C. sinensis* juvenile infection on mouse liver at transcriptomic level, cluster and enrichment analyses of DEGs were performed. A total of 3268 DEGs were detected. The heatmaps of differential expressions in different infection time points are presented in Fig. 4a. Among them, the change trend of DEGs at 3 d and 7 d time points was consistent, with the most pronounced change observed in the 7 d group. The volcano diagram analysis revealed that the DEGs of 6 h vs. 0 h, 18 h vs. 0 h, 24 h vs. 0 h, 3 d vs. 0 h and 7 d vs. 0 h were 454 (117 up- and 337 downregulated), 428 (218 up- and 210 downregulated), 235 (121 up- and 114 downregulated), 482 (308 up- and 174 downregulated) and 1669 (1314 up- and 355 downregulated), respectively (|logFC|> 1, *P* < 0.05, Additional file 2: Fig. S2). Consequently, the number of DEGs was lowest at 24 h of infection and highest on 7 d.

According to the KEGG analysis, signaling pathways related to immunity, inflammation, tumor and metabolism were significantly enriched, some of which appeared at multiple continuous or discontinuous time points and some at a single time point. Immune and inflammation-related pathways were mainly enriched in pathways of antigen processing and presentation (6 h, 24 h and 3 d), phagosome (6 h, 18 h, 3 d and 7 d), NOD-like receptor (NLR) (6 h, 18 h and 24 h), Th17 cell differentiation (18 h and 24 h), Ras (24 h), MAPK (3 d), inflammatory mediator regulation of TRP channels (7 d) and cytokine-cytokine receptor interaction (7 d). Tumor-related signaling pathways were mainly concentrated in cancer (18 h, 24 h, 3 d and 7 d), chemical carcinogenesis (18 h, 24 h and 7 d), PI3K-Akt (18 h, 24 h, 3 d and 7 d), hepatocelleular carcinoma (18 h) and p53 (3 d). Metabolism-related pathways mainly included metabolic (18 h, 24 h, 3 d and 7 d), retinol metabolism (18 h, 24 h and 3 d), PPAR (24 h and 3 d), thyroid hormone (18 h), steroid biosynthesis (18 h), drug metabolism-cytochrome P450 (18 h), linoleic acid metabolism (24 h), cholesterol metabolism (24 h) and glycine, serine and threonine metabolism (3 d). Moreover, pathways closely related to cell proliferation, differentiation and pathology were also enriched, including cell cycle (3 d and 7 d), ECM-receptor interaction (7 d) and focal adhesion (7 d) (Fig. 4b–f). In addition, the qPCR results showed that the transcriptional expression trends of key genes (Col1a1, Col1a2, Spp1, Hmmr, Cdc20 and Ccnb2) involved in the above important pathways were consistent with the RNA sequencing results, confirming the credibility of the sequencing data (Additional file 2: Fig. S3).

### DEPs annotation and functional enrichment of *C. sinensis*-infected mouse liver

**Fig. 5:**
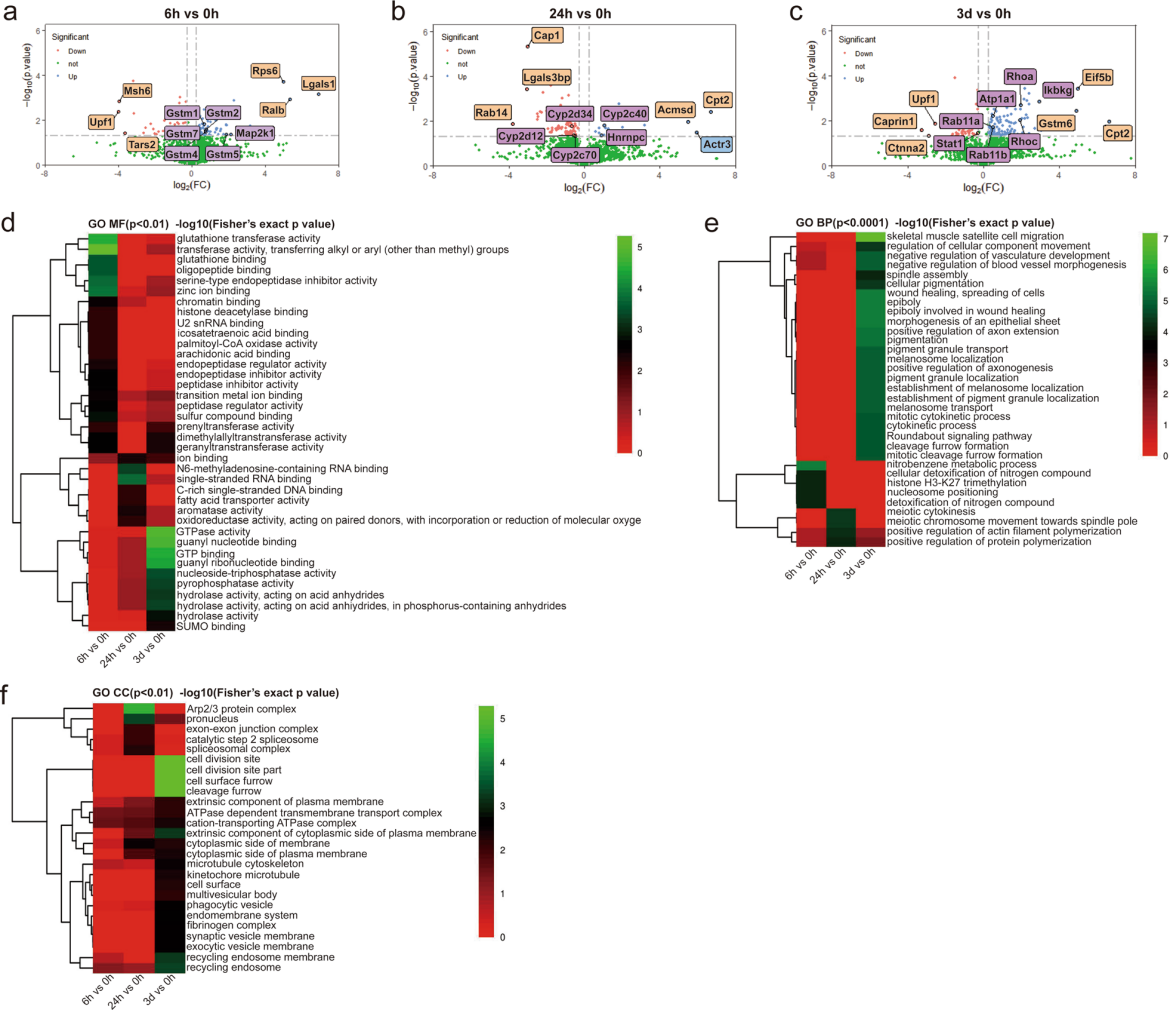
Volcano plots and GO enrichment analysis of DEPs in mouse liver at different time points post *Clonorchis sinensis* infection. Top three for each of up- and downregulated proteins and representative proteins are labeled in each volcano plot. **a** Volcano plot of DEP analysis between 6 and 0 h groups (135 up- and 87 downregulated). **b** Volcano plot of DEP analysis between 24 and 0 h groups (57 up- and 145 downregulated). **c** Volcano plot of DEP analysis between 3 d and 0 h groups (268 up- and 78 downregulated). **d** Enriched GO terms in MF. **e** Enriched GO terms in BP. **f** Enriched GO terms in CC. The more obvious the green is, the more significant the change

For insights into the liver-expressed protein altered by *C. sinensis* infection, proteome analyses of mouse livers from 0 h, 6 h, 24 h and 3 d groups were performed. The volcano analysis revealed that the DEPs of 6 h vs. 0 h, 24 h vs. 0 h and 3 d vs. 0 h were 222 (135 up- and 87 downregulated), 202 (57 up- and 145 downregulated) and 346 (268 up- and 78 downregulated) with absolute value of FC ≥ 1.2 and *P* < 0.05, respectively (Fig. 5a–c). The first six differential proteins (orange rectangles), proteins significantly involved in GO and KEGG enrichment (purple rectangles), and the protein corresponding to both conditions (blue rectangle) between each infection group and the 0 h group were marked on volcano diagrams (Fig. 5a–c).

GO analysis revealed that at 6 h post infection, changes in MF of transferase activity (mainly glutathione transferase) and BPs of nitrobenzene metabolic process and cellular detoxification of nitrogen compound were markedly triggered, primarily caused by high expression of Gstm1, Gstm2, Gstm4, Gstm5 and Gstm7 proteins (Fig. 5a, b and e). At 24 h of infection, CC of Arp2/3 protein complex involved in the BP of positive regulation of actin filament polymerization was significantly enriched, which was closely associated with the significant expression of Actr3 protein (Fig. 5b, e and f). On the 3rd day of infection, the CCs of cell division site and cell surface furrow, MFs of GTPase activity and GTP binding, and BPs of epiboly involved in wound healing, wound healing, spreading of cells and mitotic cytokinetic process were mainly triggered because of the high expression of Ras- and Rho-related proteins (e.g. Rhoa, Rhoc, Rab11a and Rab11b) (Fig. 5c–e).

### KEGG analysis of the mouse liver DEPs triggered by *C. sinensis* infection

**Fig. 6:**
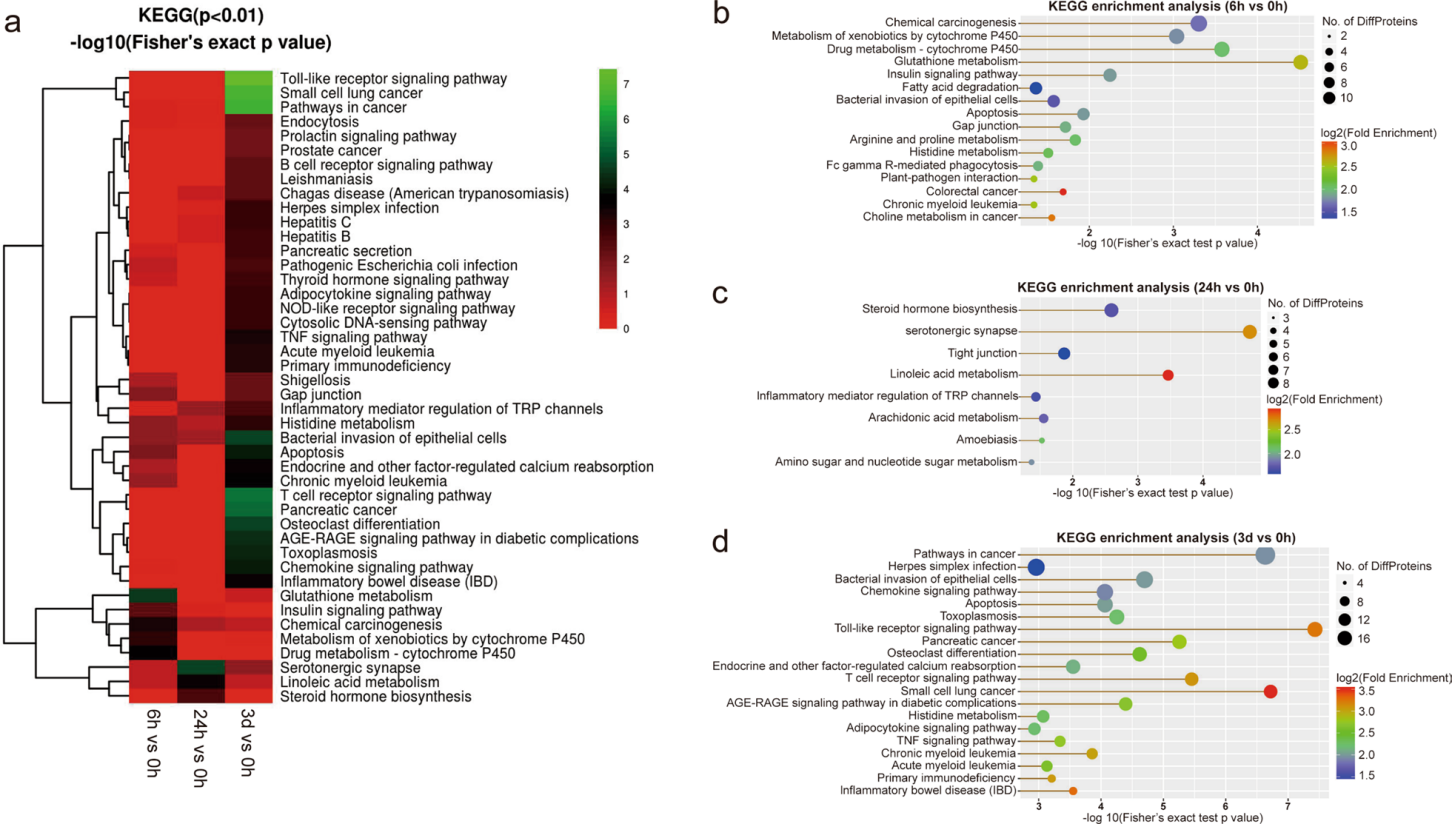
KEGG enrichment analysis of the DEPs identified in mouse liver from different infection time groups. **a** Heatmap indicating the KEGG pathway enrichment analysis of identified DEPs among 0 h, 6 h, 24 h and 3 d groups. **b** KEGG pathway enrichment analysis of DEPs between 6 and 0 h groups. **c** KEGG pathway enrichment analysis of DEPs between 24 and 0 h groups. **d** KEGG pathway enrichment analysis of DEPs between 3 d and 0 h groups

The DEPs were subjected to KEGG pathway enrichment analysis. In DEPs of 6 h vs. 0 h, the pathways of glutathione metabolism, drug metabolism-cytochrome P450, metabolism of xenobiotics by cytochrome P450, chemical carcinogenesis, apoptosis and gap junction were dominantly enriched (Fig. 6a and b). The highly enriched signaling pathways between 24 and 0 h groups included linoleic acid metabolism, serotonergic synapse, steroid hormone biosynthesis, tight junction, arachidonic acid metabolism and inflammatory mediator regulation of TRP channels (Fig. 6a and c). On 3 d post infection, immune response-related pathways were observed, including pathways of Toll-like receptor (TLR), T cell receptor (TCR), chemokine, TNF and NLR, all of which were mainly related to the significant upregulation of Ikbkg protein (NF-κB essential modulator) (Fig. 5c, Fig. 6a and d). Additionally, pathway-related cancer, apoptosis and metabolism (histidine and thyroid hormone) were also significantly enriched on the 3rd day of infection (Fig. 6a and d).

### Screening of differential metabolites in mouse liver elicited by *C. sinensis* infection

**Fig. 7:**
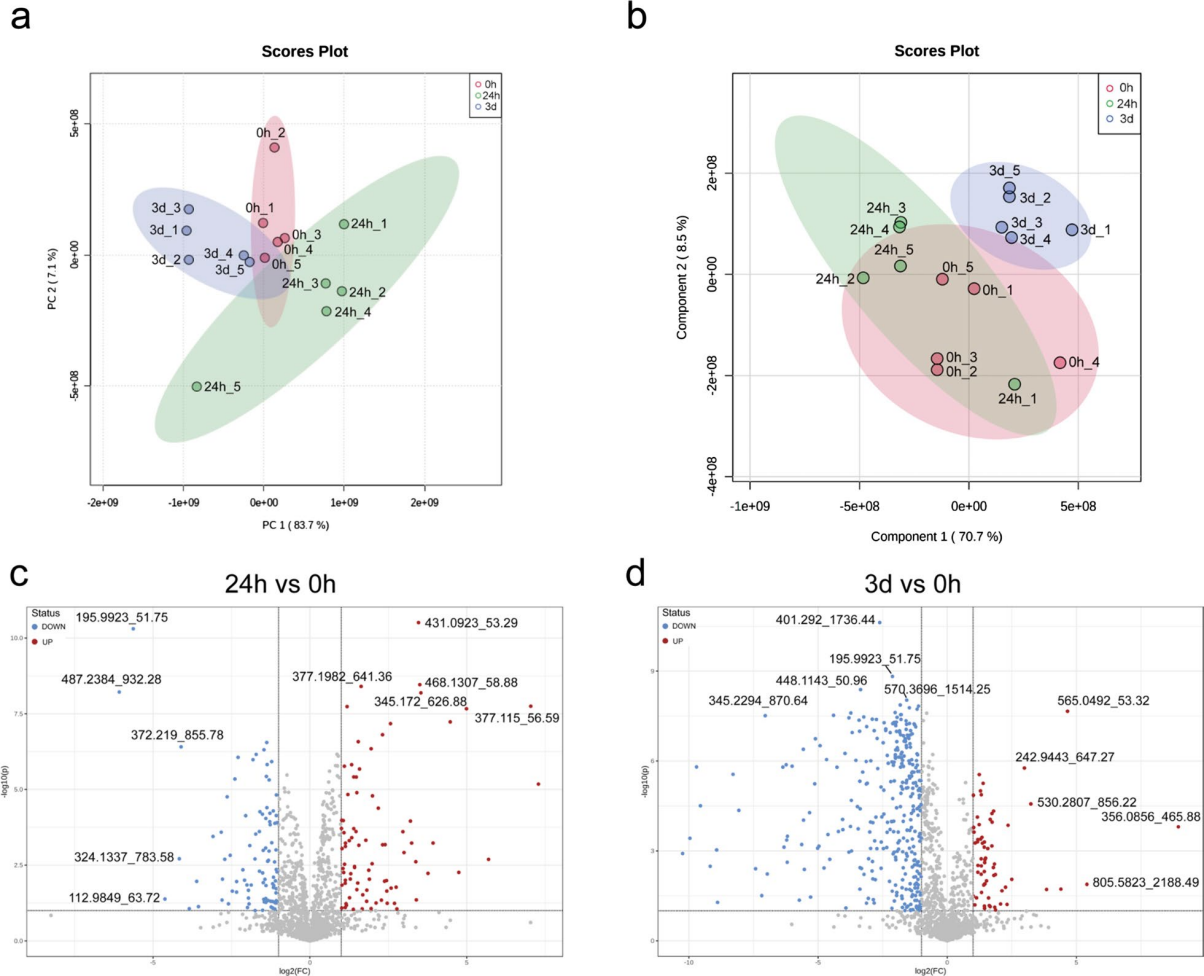
Multivariate analysis and differential metabolite analysis in negative ion mode of mouse liver from different infection time groups. The overall trend, difference degree and differential metabolites of samples were observed among groups. **a** PCA score diagram of 0 h, 24 h and 3 d groups. **b** PLS-DA score diagram of 0 h, 24 h and 3 d groups. **c** Volcano plot of metabolite change of 24 h group compared with 0 h group. **d** Volcano plot of differential metabolites of 3 d group compared with 0 h group. *PCA* Principal component analysis. *PLS-DA* Partial least square discrimination analysis

To detect metabolite changes during *C. sinensis* infection, livers from 0 h, 24 h and 3 d groups were subjected to untargeted LC-MS/MS analysis. The evaluation of three quality control contents showed that the error caused by the experimental instrument was small, and the repeatability of the experiment was excellent (Additional file 2: Fig. S4). A total of 850 metabolites were detected, of which 330 were detected in positive ion mode and 520 in negative ion mode. In the principal component analysis (PCA) and partial least squares discrimination analysis (PLS-DA) models, the samples were dispersed among groups and gathered within groups, indicating that metabolites among groups were different (Fig. 7a and b).

The volcano diagrams revealed that in negative ion mode, comparing the 24 h group and 0 h group, the metabolites of citric acid, isocitric acid, D-threo-isocitric acid, diketogulonic acid, 2,3-diketo-L-gulonate, fructosamine, beta-D-glucosamine, 2-vinyl-4H-1,3-dithiin (2VD), oxypurinol, xanthine and 6,8-dihydroxypurine were significantly upregulated, while 7-sulfocholic acid (CA7S) and other metabolites (most not included in the HMDB database) were significantly downregulated (Fig. 7c). Comparing the 3 d group and 0 h group, uridine diphosphate glucose (UDP-glucose) and uridine diphosphate galactose (UDP-galactose) were significantly upregulated, while N-acetylneuraminic acid, sciadonic acid, retinyl ester, eicosapentaenoic acid and methyltestosterone were significantly downregulated (Fig. 7d). The analysis results of the changes of liver metabolites among different groups under positive ion mode are shown in supplementary material (Additional file 2: Fig. S5).

### Hierarchical cluster and enrichment analysis of mouse liver differential metabolites evoked by *C. sinensis* infection

**Fig. 8:**
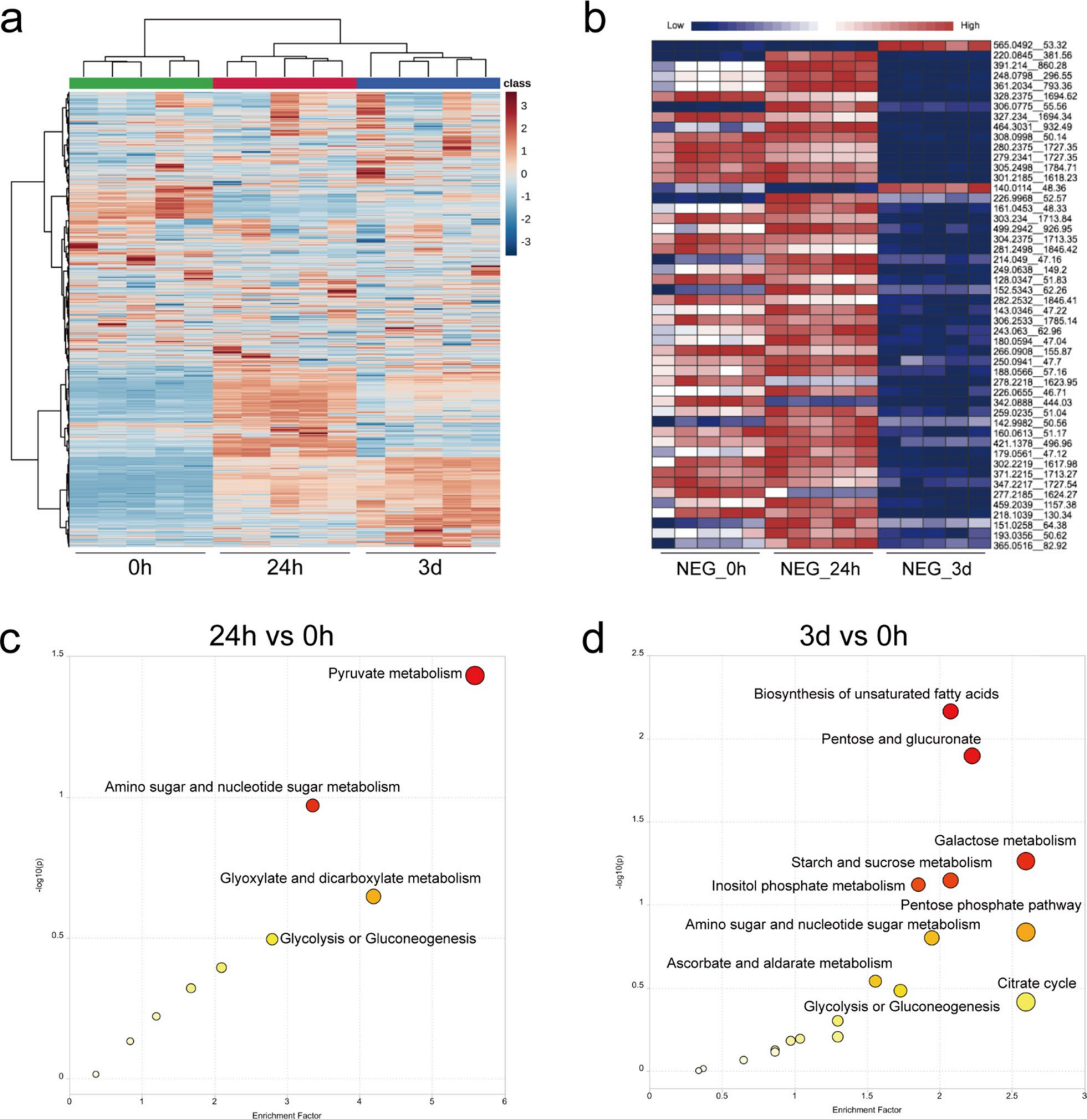
Cluster, annotation and KEGG enrichment analysis of differential metabolites of mouse liver from different time points post infection. **a** Cluster analysis of all differential metabolites. **b** Heat map of significant metabolite hierarchical clustering in the negative ion mode. **c** KEGG enrichment analysis of the differential metabolites between 24 h group and 0 h group. **d** KEGG enrichment analysis of differential metabolites between 3 d group and 0 h group

Hierarchical cluster analyses of all metabolite alterations among 0 h, 24 h and 3 d groups are shown in Fig. 8a. Similarly, significant differential metabolites (VIP > 1, *P* < 0.05) detected in negative ion mode are showed in Fig. 8b. In addition, hierarchical clustering results of the changes of liver metabolites among different groups under positive ion mode were shown in supplementary material (Additional file 2: Fig. S6).

The differential metabolites in negative and positive ion mode were merged to further perform KEGG pathway analysis. In the enrichment analysis of 24 h group vs. 0 h group, the top nine related metabolic pathways were displayed, among which pathways of pyruvate metabolism, amino sugar and nucleotide sugar metabolism, and glyoxylate and dicarboxylate metabolism were considered to be the main metabolic pathways (Fig. 8c). In the enrichment analysis of the 3 d group vs. 0 h group, the top 19 related metabolic pathways were displayed, among which pathways of biosynthesis of unsaturated fatty acids, pentose and glucuronate interconversions and galactose metabolism were considered to be the major metabolic pathways (Fig. 8d). Additionally, compared with the 0 h group, pathways of amino sugar and nucleotide sugar metabolism and glycolysis or gluconeogenesis were significantly enriched in both the 24 h and 3 d groups (Fig. 8c and d).

## Discussion

Our results showed that small white nodules were macroscopically observed in the left lobe of mouse liver from 3 d post infection. Histopathological changes, including inflammatory cell infiltration, biliary hyperplasia and collagen deposition, were observed as early as 24 h post infection. Serological test data confirmed a gradual elevation in the levels of ALT and AST, which are biomarkers of hepatocyte injury [22], starting from 24 h of infection. Furthermore, in our experiment, inflammation-related indicators in blood, such as WBC, CRP, IL-6, IL-1β and TNF-α, exhibited varying degrees of elevation since 18 h of infection. WBC and CRP are commonly used as systemic inflammatory indices [23, 24]. CRP is an acute phase plasma protein which can be activated by pro-inflammatory cytokines of IL-1β, IL-6 and TNF-α [24, 25]. Additionally, the ratio of CD4^+^/CD8^+^ T cells in peripheral blood tended to gradually decrease with prolonged infection, indicating the possibility of persistent infection [14, 26, 27]. Therefore, the above results suggested that infection by *C. sinensis* juveniles could trigger acute liver injury and systemic inflammatory responses within a very short period of time.

Next, a combination of transcriptomics, proteomics and metabolomics was used to reveal the molecular changes in the liver caused by the early infection by *C. sinensis* juveniles. By comparing the heatmap of DEGs in transcriptomics from 6 h to 7 d after infection, we found that both the 3 d and 7 d groups showed similar trends compared to the 0 h group. Therefore, selecting time points within 3 days for further proteomic and metabolomic analysis is highly representative. In general, our analyses showed that many crucial biological changes in the liver during *C. sinensis* infection were simultaneously reflected in our transcriptomic, proteomic and metabolomic results. Therefore, compared with a single-omics, our multi-omics analyses provided a more comprehensive view in the digging of key molecular events and genes involved in the infection, allowing us to better unveil its mechanism [28–30]. Briefly, our multi-omics results demonstrated that acute liver injury caused by *C. sinensis* juvenile forced the liver to activate detoxification and antioxidant defense system and respond to the injury by enhancing cell deformation, proliferation, migration and tissue repair. During this process, we observed enrichment of many immunity, inflammation, tumor and metabolism-related genes and signaling pathways.

Proteomic analysis revealed a significant enrichment of the apoptosis pathway, particularly on 3rd day post infection, implying the occurrence of cell death and liver injury [31, 32]. In addition, pathways of gap and tight junctions were enriched at 6 and 24 h, respectively, suggesting potential impacts on liver homeostasis, blood-biliary barrier and intercellular communication [33–35]. In addition, GO analysis showed that MF of glutathione transferase activity and BP of cellular detoxification of nitrogen compound were primarily concentrated at 6 h of infection. Correspondingly, KEGG analysis at this time mainly focused on glutathione metabolism and cytochrome P450-related pathways. The upregulated proteins involved in the aforementioned MF, BP and pathways were detoxifying enzymes of Gstm1, Gstm2, Gstm4, Gstm5 and Gstm7, which can protect against exogenous toxins and endogenous oxidative stress [36]. Moreover, transcriptomic data also showed significant enrichment of cytochrome P450 pathway at 18 h of infection. Cytochrome P450 enzymes are the most abundantly expressed in hepatocytes, where they play important roles in metabolizing xenobiotics and regulating intracellular stress responses [37]. Glutathione, the most common small-molecule antioxidant in hepatocytes, participates in important physiological processes such as free radical scavenging, antioxidant and detoxification [38]. Based on these findings, we hypothesized that the host liver rapidly initiates the detoxification and antioxidant defenses against acute injury caused by infection by *C. sinensis* juveniles.

Proteomic data of 24 h showed that CC of Arp2/3 protein complex and BP of positive regulation of actin filament polymerization were obviously enriched, indicating the occurrence of cell deformation, migration and proliferation in the liver [39]. At 3 d post infection, the CC of cell division site, MF of GTPase activity and the BPs of epiboly involved in wound healing and mitotic cytokinetic process were dominantly activated. Among them, Ras- and Rho-related proteins were significantly upregulated. Previous studies have shown that Rho GTPases are crucial regulators of actin cytoskeleton and affect multiple biological functions, including cell migration, division and wound healing [40, 41]. Jiang et al. demonstrated that Ras GTPases may play important roles in the regulation of cell cycle and immune-related pathways [42]. Consistently, transcriptomic analysis revealed significant enrichment of the cell cycle pathway at both 3 and 7 d post infection. Thus, infection of *C. sinensis* juveniles triggered biological processes involving cell division, migration and wound healing in the host liver.

Both proteomic and transcriptomic analysis confirmed that *C. sinensis* juvenile infection stimulated the enrichment of multiple immune, inflammatory and tumor-related signaling pathways in the liver. The pathways of NLR and inflammatory mediator regulation of TRP channels were significantly enriched in bi-omics analyses. NLRs, as the major cytosolic pattern recognition receptors (PRRs) for innate immunity, are critical intracellular sensors for host defense against bacteria, viruses and parasites. Moreover, NLRs drive inflammatory responses through activation of MAPK and NF-κB signaling pathways [43, 44]. There is a wealth of evidence suggesting that TRP channels such as TRPV1, TRPV3 and TRPM8 may play vital roles in the progression of fibroproliferative diseases in the lung, liver and heart and promote both acute and chronic inflammatory processes [45–47]. Proteomic results showed that pathways of TLR, TCR, TNF, cancer and chemokine were significantly enriched on the 3rd day of infection, all of which were associated with upregulation of Ikbkg. Ikbkg (also known as IKKγ) is essential for rapid activation of NF-κB by pro-inflammatory signaling cascades [48]. Previous reports have confirmed the upregulation of both TLR2 and TLR4 during *C. sinensis* infection, and TLR4 can promote pathogen-associated biliary fibrosis [49, 50]. Additionally, KEGG analysis of transcriptomics also revealed the enrichment of numerous immune-inflammatory and tumor-related pathways, such as Th17 cell differentiation, PI3K-Akt, MAPK, p53 and cancer. The PI3K/Akt/mTOR pathway has been reported to be overexpressed in nearly 50% of hepatocellular carcinomas, and it plays a crucial role in tumorigenesis and progression [51].

Additionally, the three omics analyses collectively showed that early infection by *C. sinensis* juveniles had significant impacts on the metabolism of lipids, carbohydrates and amino acids in the liver. Transcriptomic results proved that the metabolic pathway was significantly enriched from 18 h to 7 d after infection with the largest input number of DEPs, especially at the 24 h point. At the 24 h time point, proteomic and transcriptomic results jointly showed a significant enrichment of linoleic acid, arachidonic acid and cholesterol metabolism and steroid hormone biosynthesis pathways. Proteomic and metabolomic data collectively displayed that the pathway of amino sugar and nucleotide sugar metabolism was significantly enriched at 24 h and 3 d post infection. Furthermore, transcriptomic analysis enriched the glycine, serine and threonine and retinol metabolism pathways at the 3 d time point, proteomic analysis enriched the histidine (6 h and 3 d) and arginine and proline (6 h) metabolism pathways, and metabolomic analysis enriched pathways of pyruvate metabolism (24 h), glycolysis or gluconeogenesis (24 h and 3 d) and biosynthesis of unsaturated fatty acids (3 d). Moreover, both transcriptomic and proteomic data revealed a significant enrichment of pathways regulating liver metabolism, such as PPAR and thyroid hormone, upon stimulation by *C. sinensis*. PPAR is critical for hepatic lipid metabolism [52]. Thyroid hormone is essential for maintaining hepatic metabolic homeostasis and normal body development [53, 54].

Besides, the metabolomic analysis identified several functional metabolites. Metabolomic data displayed that at 24 h post infection, CA7S, a bile acid metabolite, was significantly downregulated, whereas metabolites of glucosamine and 2VD were significantly upregulated. CA7S has been reported as an agonist of Tgr5 and can increase the expression of Tgr5, thereby preventing cholestasis and suppressing the inflammatory responses [55–57]. Glucosamine, as an amine sugar, has been reported to stimulate immune cells and mediate immune function by activating NF-κB, p38 and PKA pathways [58]. It was reported that 2VD could reduce intracellular ROS and increase total antioxidant status in vivo [59]. At 3 d post infection, metabolites of UDP-glucose and UDP-galactose were upregulated; both are crucial extracellular signaling molecules acting as potent agonists of extensively expressed P2Y14 receptor [60]. It has been documented that P2Y14 not only plays an important role in the immune and inflammatory responses but also promotes toxicity and hepatobiliary fibrosis [60, 61].

Different developmental stages of *C. sinensis* stimulate the host to generate complex and specific immune responses, characterized by a predominance of type 1 immune response in the early stage of infection and a predominance of type 2 immune response in the late stage of infection [13, 14]. Our study showed that systemic acute inflammatory responses and progressive liver injury were induced within 7 days of *C. sinensis* juvenile infection. In addition to abundant enrichment of immune-inflammatory pathways, the liver transcriptomics results verified that fibrosis-related pathways such as ECM-receptor interaction and focal adhesion were also significantly observed on the 7th day of infection [62, 63]. However, Zhang et al. detected increased hepatic Th2 and Treg subsets in different strains of mice infected with *C. sinensis* for 28 days, presumably strongly associated with biliary periductal fibrosis [64]. Furthermore, Kong et al. revealed that an increased Treg/Th17 ratio during the late stage of infection was conducive to the pathogenicity of *C. sinensis* [65]. Therefore, as the infection progresses, the host undergoes a switch from pro- to anti-inflammatory responses, which further contributes to the survival and pathogenesis of parasites. However, the specific immune switching mechanism requires further in-depth study.

## Conclusion

In summary, early infection by *C. sinensis* juveniles could rapidly trigger an acute systemic inflammatory response and cause liver injury and hepatobiliary lesions in the host. The multi-omics analysis suggested that the liver would quickly initiate detoxification and antioxidant stress response systems to combat the acute damage. In addition, biological processes such as cell apoptosis, deformation, division, migration, connection change and wound healing were obviously induced during the early stage of worm infection. Furthermore, KEGG analysis of multi-omics showed numerous immune, inflammation, fibrosis, tumor and metabolism-related signaling pathways enriched in the liver. In general, our research reveals the mechanism of early infection of *C. sinensis* in the host from multiple perspectives, which provides valuable insights into the study of the *C. sinensis*-host interaction.

### Supplementary Information

**Additional file 1: Table S1.** List of primer sequences used for qPCR.

**Additional file 2: Figure S1.** Proportion of CD4^+^ and CD8^+^ T cells in peripheral blood from different groups. (a) Percentages of CD4^+^ T cells and CD8^+^ T cells in peripheral blood. (b) Statistical analysis of the frequency of CD4^+^ T cells. (c) Statistical analysis of the frequency of CD8^+^ T cells. (d) Statistical analysis of CD4^+^/CD8^+^ T cell ratio. Data are shown as mean ± SD. *P < 0.05. **Figure S2.** Volcano plots of differential gene expression analysis of mouse liver from different groups. Up- and downregulated genes are highlighted in red and blue, respectively. (a) 454 DEGs (117 up and 337 down) were found between 6 h group and 0 h group. (b) 428 DEGs (218 up and 210 down) were found between 18 h group and 0 h group. (c) 235 DEGs (121 up and 114 down) were found between 24 h group and 0 h group. (d) 482 DEGs (308 up and 174 down) were found between 3 d group and 0 h group. (e) 1696 DEGs (1341 up and 355 down) were found between 7 d group and 0 h group. **Figure S3.** The mRNA expression of Col1al, Col1a2, Spp1, Hmmr, Cdc20 and Ccnb2 at different time points. The fold change of mRNA expression levels of Col1al (a), Col1a2 (b), Spp1 (c), Hmmr (d), Cdc20 (e) and Ccnb2 (f) at 0 h, 6 h, 24 h, 3 d and 7 d. **Figure S4.** Quality control and TIC chart of raw data for metabolic analysis. The stability of the instrument, repeatability of the experiment and reliability of the data quality were comprehensively evaluated. (a–b) Comparison of spectral overlap of total ion chromatogram (TIC) of QC samples. (c–d) The peaks extracted from all experimental samples and QC samples were analyzed with principal component analysis (PCA). (e–f) Retention time deviation between the control group and the experimental groups in the negative and positive ion mode. **Figure S5.** Multivariate analysis and differential metabolite analysis in positive ion mode. (a) Principal component analysis (PCA) score diagram of 0 h, 24 h and 3 d groups. (b) Partial least squares discrimination analysis (PLS-DA) score diagram of 0 h, 24 h and 3 d groups. (c) Volcano plot of metabolites change of 24 h group compared with 0 h group. (d) Volcano plot of differential metabolites of 3 d group compared with 0 h group. **Figure S6.** Heatmap of hierarchical clustering of significantly different metabolites in positive ion mode.

## Data Availability

The raw RNA-seq data have been deposited in the NCBI database under the accession number PRJNA917789. The mass spectrometry proteomics data have been deposited in the Proteome Xchange Consortium via the iProX partner repository with the dataset identifier PXD039310.

## Abbreviations

*C. sinensis*: *Clonorchis sinensis*
WHO: World Health Organization
ESPs: Excretory-secretory products
ALT: Alanine aminotransferase
AST: Aspartate aminotransferase
WBC: White blood cell
LYM: Lymphocyte
GRAN: Granulocyte
MONO: Monocyte
RBC: Red blood cell
HCT: Hematocrit
HGB: Hemoglobin
PLT: Platelet
CRP: C-reactive protein
GO: Gene ontology
KEGG: Kyoto Encyclopedia of Genes and Genomes
DEGs: Differentially expressed genes
DEPs: Differentially expressed proteins
LC–MS/MS: Liquid chromatography tandem mass spectrometry
NLR: NOD-like receptor
FC: Fold change
MF: Molecular function
BP: Biological process
CC: Cell component
TLR: Toll-like receptor
TCR: T cell receptor
PCA: Principal component analysis
PLS-DA: Partial least squares discrimination analysis
2VD: 2-Vinyl-4H-1,3-dithiin
CA7S: 7-Sulfocholic acid
UDP-glucose: Uridine diphosphate glucose
UDP-galactose: Uridine diphosphate galactose
HSCs: Hematopoietic stem cells
