# Supplementary material for: Multilayer omics reveals the molecular mechanism of early infection of Clonorchis sinensis juvenile
Source: Parasit Vectors. 2023 Aug 16;16:285. doi: 10.1186/s13071-023-05891-1 (PMC10428567; doi:10.1186/s13071-023-05891-1)
Supplement: Supplementary file 1 — Additional file 1: Table S1. List of primer sequences used for qPCR. [file 13071_2023_5891_MOESM1_ESM.docx]

**Additional file 1.**

**Table S1. List of primer sequences used for qPCR.**

| Gene | Forward (5’- 3’) | Reverse (5’- 3’) |
| --- | --- | --- |
| Col1a1 | GACGCCATCAAGGTCTACTGC | GGAAGGTCAGCTGGATAGCG |
| Col1a2 | AAGGATACAGTGGATTGCAGG | TCTACCATCTTTGCCAACGG |
| Spp1 | AGAGCGGTGAGTCTAAGGAGT | TGCCCTTTCCGTTGTTGTCC |
| Hmmr | AGCAGAAGGAGGAGCAGAGTG | TTGGGCGTGAGCAGCAATATG |
| Cdc20 | GCCCACCAAAAAGGAGCATC | ATTCTGAGGTTTGCCGCTGA |
| Ccnb2 | AGCTCCCAAGGATCGTCCTC | TGTCCTCGTTATCTATGTCCTCG |
| β-actin | CGCTGTATTCCCCTCCATCG | CCAGTTGGTAACAATGCCATGT |
