# Supplementary material for: Multilayer omics reveals the molecular mechanism of early infection of Clonorchis sinensis juvenile
Source: Parasit Vectors. 2023 Aug 16;16:285. doi: 10.1186/s13071-023-05891-1 (PMC10428567; doi:10.1186/s13071-023-05891-1)
Supplement: Supplementary file 2 — Additional file 2: Figure S1. Proportion of CD4+ and CD8+ T cells in peripheral blood from different groups. (a) Percentages of CD4+ T cells and CD8+ T cells in peripheral blood. (b) Statistical analysis of the frequency of CD4+ T cells. (c) Statistical analysis of the frequency of CD8+ T cells. (d) Statistical analysis of CD4+/CD8+ T cell ratio. Data are shown as mean ± SD. *P < 0.05. Figure S2. Volcano plots of differential gene expression analysis of mouse liver from different groups. Up- and downregulated genes are highlighted in red and blue, respectively. (a) 454 DEGs (117 up and 337 down) were found between 6 h group and 0 h group. (b) 428 DEGs (218 up and 210 down) were found between 18 h group and 0 h group. (c) 235 DEGs (121 up and 114 down) were found between 24 h group and 0 h group. (d) 482 DEGs (308 up and 174 down) were found between 3 d group and 0 h group. (e) 1696 DEGs (1341 up and 355 down) were found between 7 d group and 0 h group. Figure S3. The mRNA expression of Col1al, Col1a2, Spp1, Hmmr, Cdc20 and Ccnb2 at different time points. The fold change of mRNA expression levels of Col1al (a), Col1a2 (b), Spp1 (c), Hmmr (d), Cdc20 (e) and Ccnb2 (f) at 0 h, 6 h, 24 h, 3 d and 7 d. Figure S4. Quality control and TIC chart of raw data for metabolic analysis. The stability of the instrument, repeatability of the experiment and reliability of the data quality were comprehensively evaluated. (a–b) Comparison of spectral overlap of total ion chromatogram (TIC) of QC samples. (c–d) The peaks extracted from all experimental samples and QC samples were analyzed with principal component analysis (PCA). (e–f) Retention time deviation between the control group and the experimental groups in the negative and positive ion mode. Figure S5. Multivariate analysis and differential metabolite analysis in positive ion mode. (a) Principal component analysis (PCA) score diagram of 0 h, 24 h and 3 d groups. (b) Partial least squares discrimination analysis (PLS-DA) score d [file 13071_2023_5891_MOESM2_ESM.docx]

**Additional file 2.**


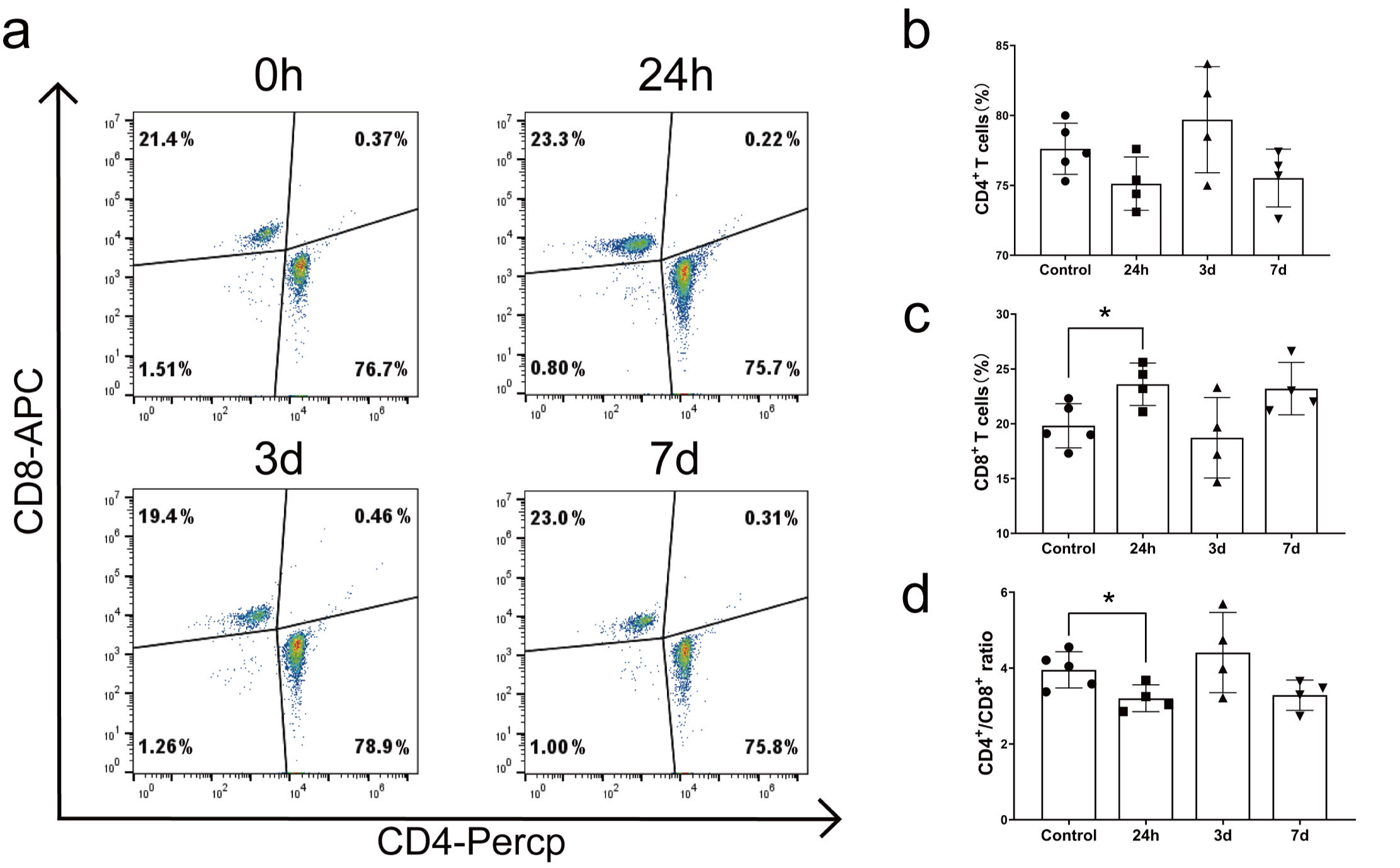


**Figure S1.** Proportion of CD4^+^ and CD8^+^ T cells in peripheral blood from different groups. (**a**) Percentages of CD4^+^ T cells and CD8^+^ T cells in peripheral blood. (**b**) Statistical analysis of the frequency of CD4^+^ T cells. (**c**) Statistical analysis of the frequency of CD8^+^ T cells. (**d**) Statistical analysis of CD4^+^/CD8^+^ T cell ratio. Data are shown as mean ± SD. **P* < 0.05.


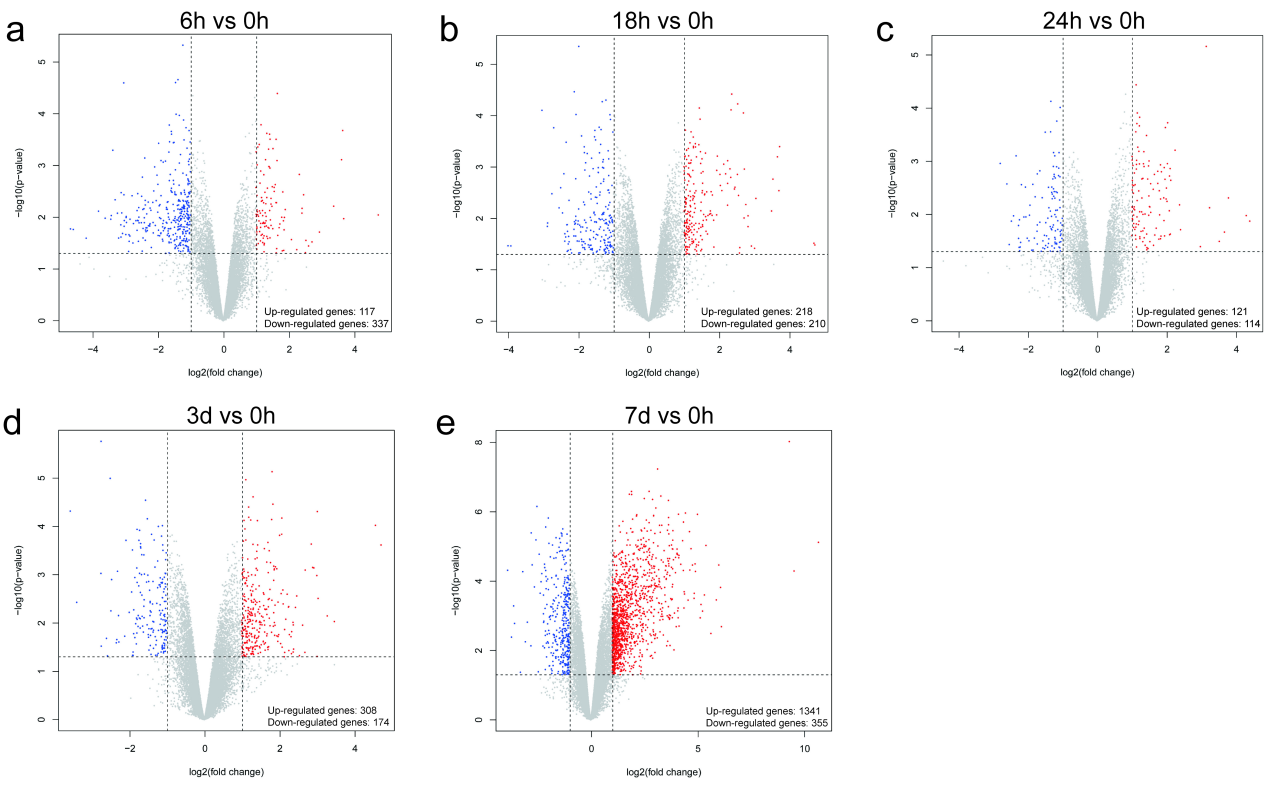


**Figure S2.** Volcano plots of differential gene expression analysis of mouse liver from different groups. Up-regulated and down-regulated genes are highlighted in red or blue, respectively. (**a**) 454 differentially expressed genes (DEGs) (117 up and 337 down) were found between 6 h group and 0 h group. (**b**) 428 DEGs (218 up and 210 down) were found between 18 h group and 0 h group. (**c**) 235 DEGs (121 up and 114 down) were found between 24 h group and 0 h group. (**d**) 482 DEGs (308 up and 174 down) were found between 3 d group and 0 h group. (**e**) 1696 DEGs (1341 up and 355 down) were found between 7 d group and 0 h group.

**
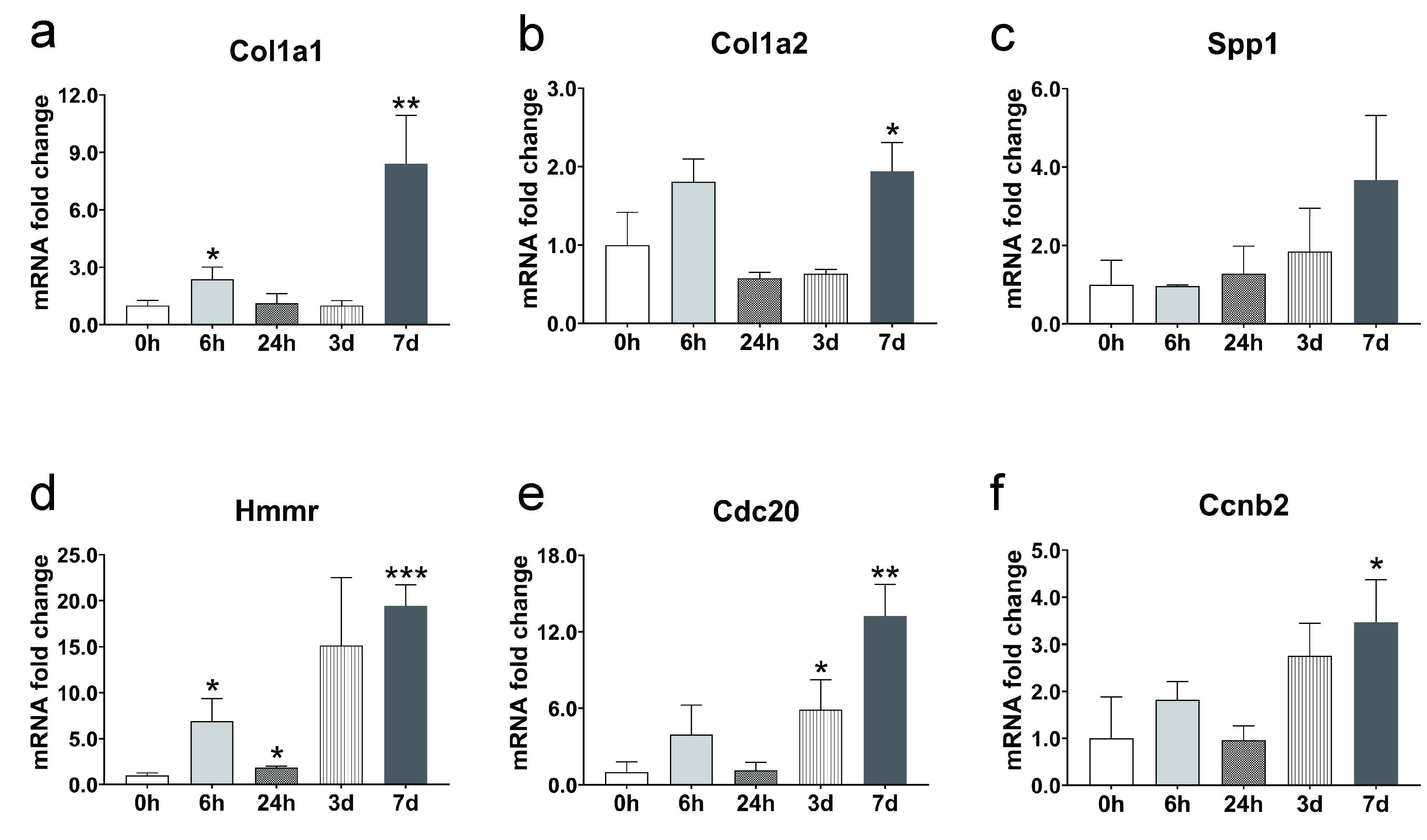
**

**Figure S3.** The mRNA expression of Col1a1, Col1a2, Spp1, Hmmr, Cdc20 and Ccnb2 at different time points. The fold change of mRNA expression levels of Col1a1 (**a**), Col1a2 (**b**), Spp1 (**c**), Hmmr (**d**), Cdc20 (**e**) and Ccnb2 (**f**) at 0 h, 6 h, 24 h, 3 d and 7 d.


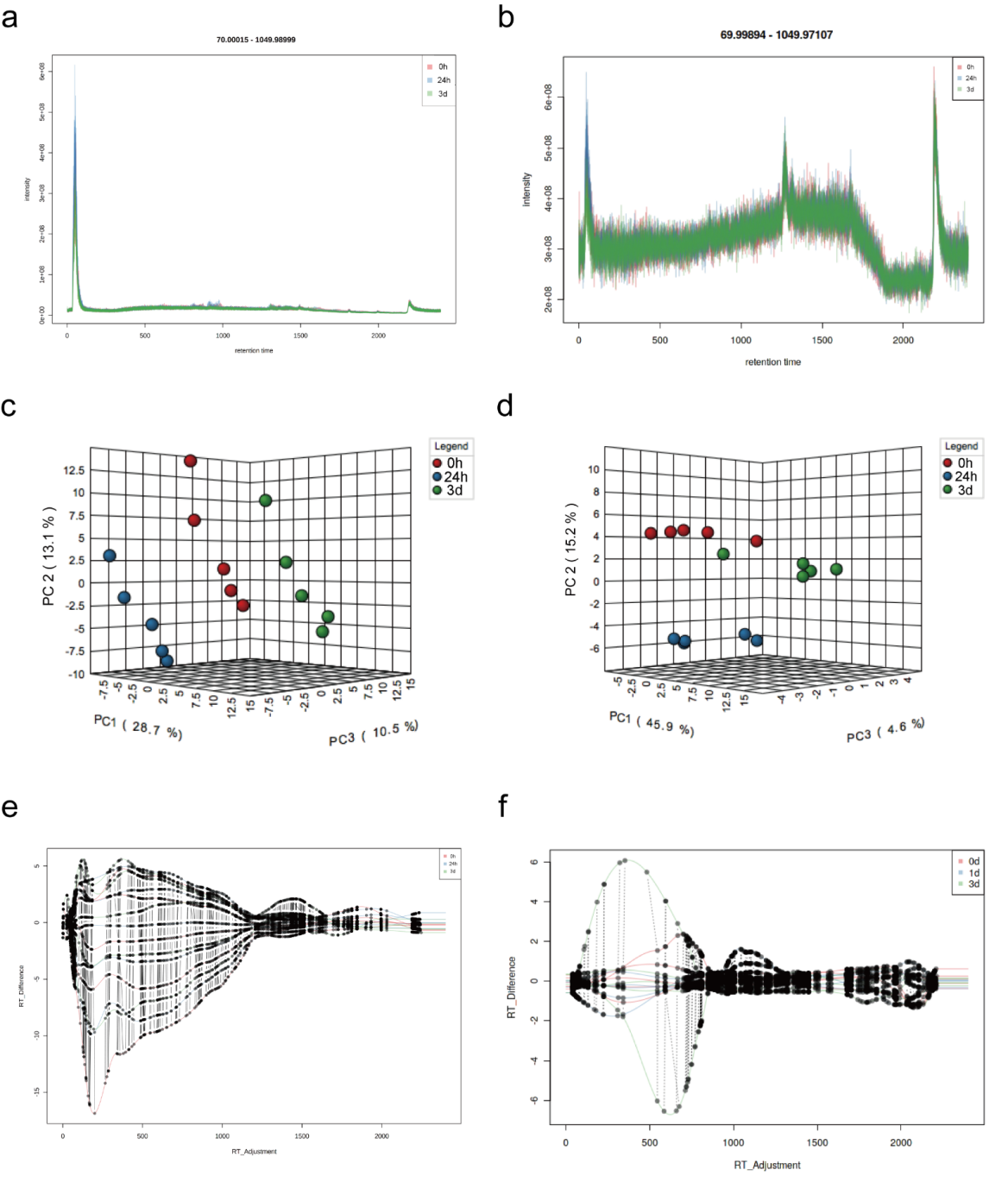


**Figure S4.** Quality control and TIC chart of raw data for metabolic analysis. The stability of the instrument, the repeatability of the experiment and the reliability of the data quality were comprehensively evaluated. (**a**-**b**) Comparison of spectral overlap of total ion chromatogram (TIC) of QC samples. (**c**-**d**) The peaks extracted from all experimental samples and QC samples were analyzed with principal component analysis (PCA). (**e**-**f**) Retention time deviation between the control group and the experimental groups in the negative and positive ion mode.


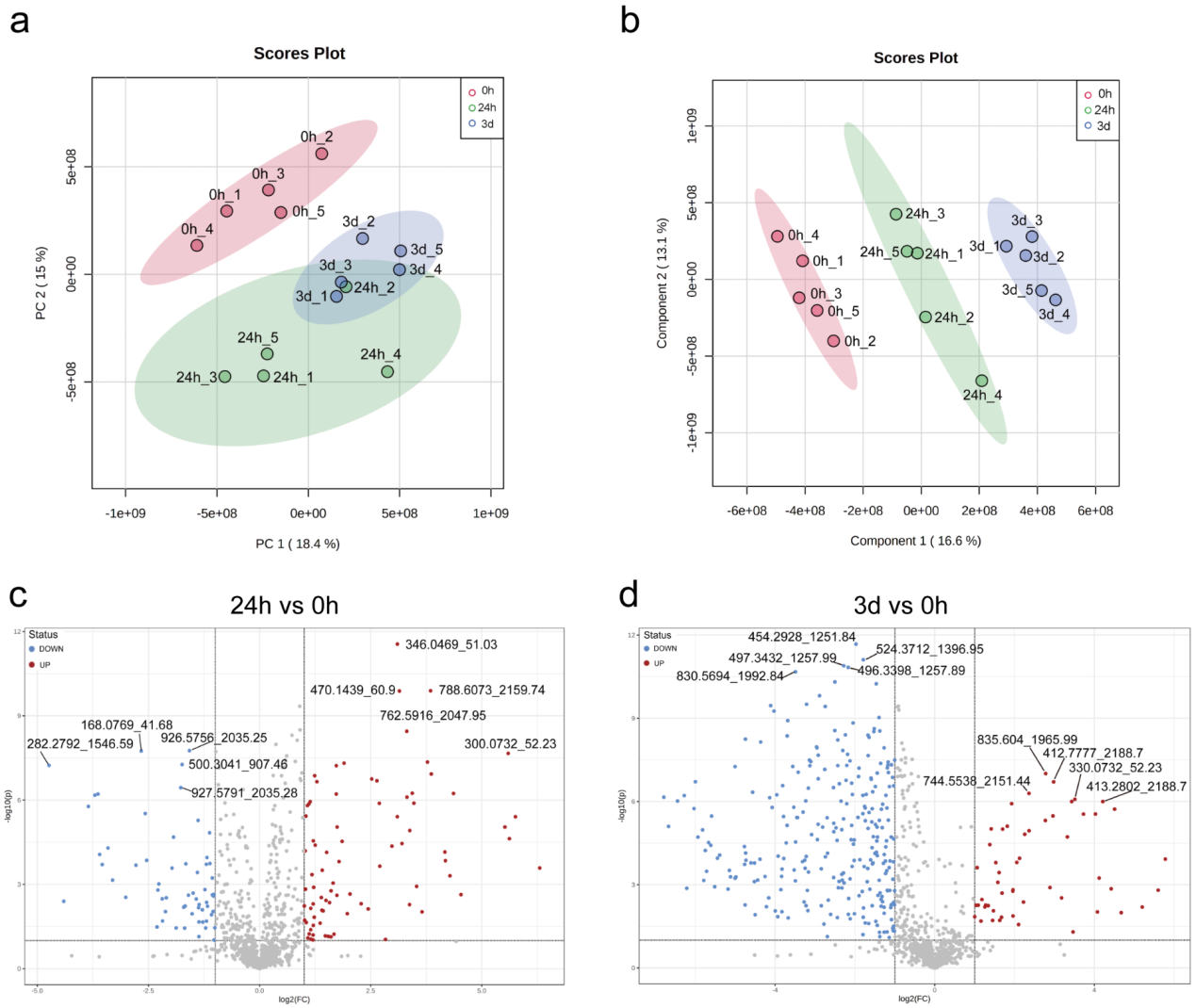


**Figure S5.** Multivariate analysis and differential metabolite analysis in positive ion mode. (**a**) Principal component analysis (PCA) score diagram of 0 h, 24 h and 3 d groups. (**b**) Partial least squares discrimination analysis (PLS-DA) score diagram of 0 h, 24 h and 3 d groups. (**c**) Volcano plot of metabolites change of 24 h group compared with 0 h group. (**d**) Volcano plot of differential metabolites of 3 d group compared with 0 h group.


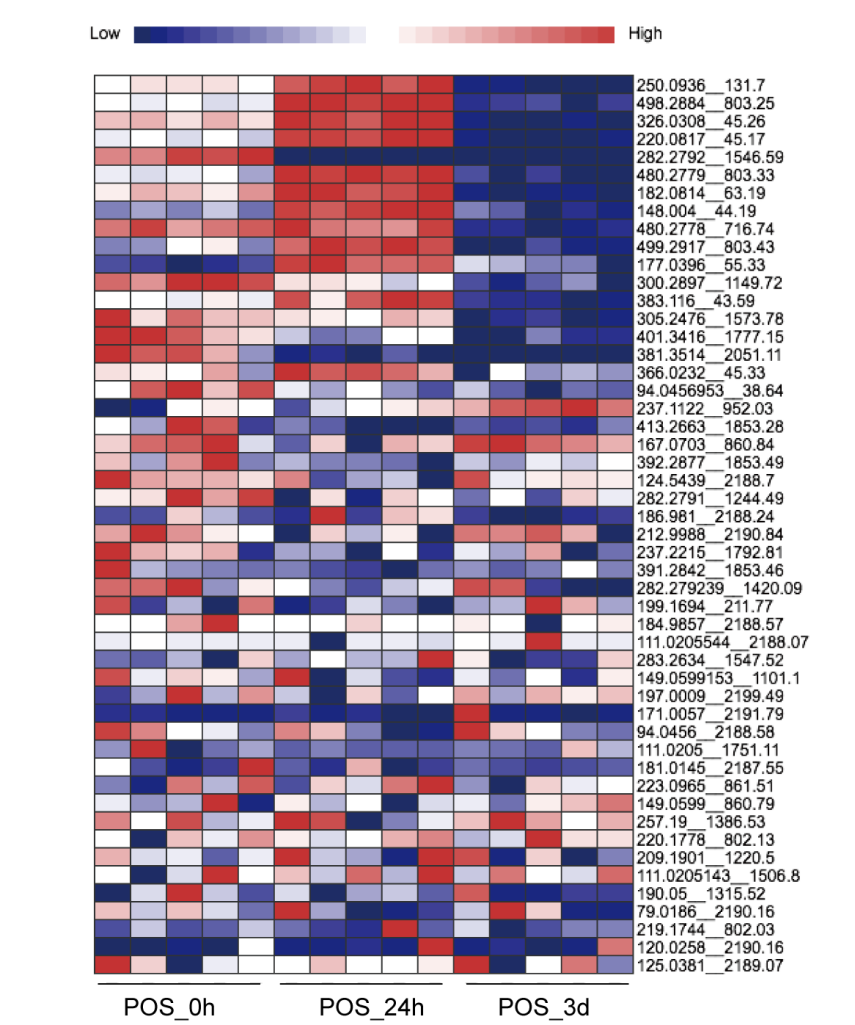


**Figure S6.** Heatmap of hierarchical clustering of significantly different metabolites in positive ion mode.
